# Two-Peg Cementless Trabecular Metal Modular Tibial Components in Total Knee Arthroplasty: A Single-Center Comparative Study with Cemented Counterparts

**DOI:** 10.3390/medicina62020296

**Published:** 2026-02-02

**Authors:** Samo Karel Fokter, Žiga Godicelj, Jure Kastelic, Vesna Levašič

**Affiliations:** 1 Clinical Department of Orthopaedics, University Medical Centre Maribor, 2000 Maribor, Slovenia; 2 Faculty of Medicine, University of Maribor, 2000 Maribor, Slovenia; ziga.godicelj@student.um.si (Ž.G.); jurekastelic05@gmail.com (J.K.); 3 National Arthroplasty Registry of Slovenia, Valdoltra Orthopaedic Hospital, 6280 Ankaran, Slovenia; vesna.levasic@ob-valdoltra.si

**Keywords:** knee, arthroplasty, total knee arthroplasty, porous tantalum, implant failure, aseptic loosening, revision surgery

## Abstract

*Background and Objectives*: Cementless total knee arthroplasty (TKA) with two-pegged Trabecular Metal (TM) tibial components composed of porous tantalum was introduced to improve fixation and reduce aseptic implant failure, particularly in younger, more active patients. Despite these theoretical advantages, mid-term outcomes remain uncertain. This retrospective study compares the survival of consecutive cementless TKAs with TM posterior-stabilized (PS) and cruciate-retaining (CR) modular tibial components with cemented PS and CR components from the same implant system, using revision for aseptic causes as the primary endpoint. *Materials and Methods*: Institutional review board approval was obtained, and a minimum follow-up of two years was required. Between January 2017 and November 2021, a total of 1202 TKAs of a single implant design were performed at a high-volume academic center by five board-certified orthopedic surgeons, predominantly for primary osteoarthritis. Of these, 826 (68.7%) procedures were cemented, and 376 (31.3%) were cementless. Demographic data and revision outcomes were collected for all patients. *Results*: There was no statistically significant difference between cohorts in the 7-year survival rate for all causes of revision (97.4%, 95% CI 95.6–99.2 for cementless vs. 97.8%, 95% CI 96.6–99.0 for cemented; *p* = 0.63). However, the 7-year survival rate for aseptic causes was significantly lower (*p* < 0.05) in the cementless TKA group (97.9%, 95% CI 96.3–99.5) compared with the cemented group (99.4%, 95% CI 98.8–100). Revisions for aseptic causes occurred in 7 cementless (1.86%) and 4 cemented (0.84%) TKAs (*p* < 0.05), most commonly due to loosening of the tibial TM component (6 [1.59%] vs. 2 [0.24%], respectively; *p* < 0.05). During follow-up, 94 patients (8.7%) died of causes unrelated to TKA. *Conclusions*: Cementless TM PS and CR modular TKAs demonstrated inferior mid-term performance compared with their cemented counterparts for aseptic reasons. These findings raise concerns regarding the durability of this cementless design and underscore the need for cautious use and further investigation.

## 1. Introduction

Fixation of tibial components remains a recognized weak point in total knee arthroplasty (TKA), primarily due to the limited bone–implant contact area, with aseptic loosening representing a major cause of revision surgery. Tibial fixation can be achieved using cemented techniques, most commonly polymethylmethacrylate (PMMA), or cementless fixation relying on bone ingrowth onto the implant surface. While both approaches offer specific advantages, as noted by Wilczyński et al., implant survival and revision rates remain the most clinically relevant outcome measures [1]. Radiostereophotogrammetric analysis (RSA) studies have demonstrated continuous migration of cemented tibial components with PMMA, reflecting ongoing bone resorption at the cement–bone interface [2,3]. To address this limitation, cementless fixation strategies aiming to achieve early mechanical stability and promote osseointegration have been developed. Although cement fixation provides superior initial fixation strength compared with cementless techniques, the surface characteristics of cementless implants are critical for supporting early bone ingrowth [4]. Among the various porous materials designed to enhance biological fixation, porous tantalum (Trabecular Metal, TM) has attracted particular attention because of its high volumetric porosity, favorable frictional properties, excellent corrosion resistance, biocompatibility, and elastic modulus closely approximating that of cancellous bone [5,6].

The TM tibial component was introduced in 2002 as a monoblock design consisting of a metal baseplate with two hexagonal pegs and an ultrahigh-molecular-weight polyethylene (UHMWPE) articulating surface, as part of the NexGen Legacy TKA system (Zimmer Biomet, Warsaw, IN, USA) [7]. Several studies have suggested that TM tibial components better preserve tibial bone mineral density (BMD) than cemented tibial implants following TKA [8,9]. In a randomized controlled trial, Fernández-Fairen et al. reported slightly superior clinical outcomes, assessed using the Knee Society Score (KSS) and the Western Ontario and McMaster Universities Osteoarthritis Index (WOMAC), in patients receiving cementless TM tibial components compared with conventional cemented implants in posterior cruciate-retaining (CR) TKA [10]. In addition, a Finnish registry study reported a 100% survival rate at seven years in 1143 TKAs using cementless TM monoblock tibial components, with revision for aseptic loosening as the primary endpoint [11].

In 2007, the manufacturer modified the original monoblock two-peg tibial component to a modular design, incorporating a titanium alloy (Ti6Al4V) baseplate coated with TM to allow the use of standard modular UHMWPE inserts of varying thicknesses. A small circular peg (central boss) was added to the anterior central region of the baseplate to accommodate the bore for a lock-down screw required to stabilize thicker polyethylene inserts. This design necessitates the creation of three holes in the proximal tibia, thereby reducing the direct contact area between the tibial baseplate and bone. Although the modular design could theoretically alter tibial component stiffness, no reduction in bone ingrowth compared with the monoblock design has been reported [12]. In a prospective randomized trial with five-year follow-up, Fricka et al. demonstrated comparable patient-reported outcomes and survivorship between cementless and cemented TKAs [13]. However, a recent BMD study by Hayakawa et al. showed that bone filling of tibial peg holes in modular tibial components increases initial fixation strength, with potential implications for long-term implant stability [14]. To date, only relatively small series, each including fewer than 1000 patients, evaluating modular TM two-peg tibial trays have been reported. These include short-term results of the Persona system (Zimmer Biomet), the successor to the NexGen Legacy system, using medial congruent (MC), ultra-congruent (UC), or CR polyethylene inserts [15], as well as a study reporting mid-term outcomes of the NexGen Legacy system limited to posterior-stabilized (PS) designs in patients with primary osteoarthritis [16]. In contrast, evidence on the mid-term performance of cementless TM modular tibial components in larger, unselected patient populations remains limited. Therefore, this retrospective study compares the survival of all consecutive cementless TKAs using TM PS and CR modular tibial components with cemented PS and CR components from the same implant system, using revision for aseptic causes as the primary endpoint.

## 2. Materials and Methods

Following approval by the local institutional review board, this retrospective study included all adult patients who underwent either cemented or cementless primary TKA using the specified implant systems for standard clinical indications at a high-volume academic center within the national public healthcare system. All primary TKAs performed between 3 January 2017 and 21 November 2021 were identified, and revision events were tracked through 31 December 2023, allowing for extended follow-up and comprehensive assessment of implant survival. This observation period was selected because both cemented and cementless implant options were concurrently available, providing a consistent basis for comparison.

Data were retrieved from the institution’s digital medical archives, which contain detailed demographic, surgical, and implant-related information. In total, 1076 patients undergoing 1202 primary TKA procedures were identified. Only primary TKA procedures in adult patients (age > 18 years) were included. Revision TKAs, cases with postoperative infection, and knees with prior implant-related procedures were excluded. Implant selection was limited to PS or CR designs according to surgeon preference, while the choice between cemented and cementless fixation was based on intraoperative bone quality. This was evaluated by visual and tactile assessment of the cancellous bone at the tibial and femoral cut surfaces, with particular attention to trabecular structure and the ability of the bone to securely hold trial components without excessive deformation or collapse. Adequate bone quality favored cementless fixation, whereas inferior bone quality led to cemented implantation. Bilateral TKA was performed in 126 patients (11.7%); of these, 86 patients received cemented implants in both knees, 28 received cementless implants bilaterally, and 12 patients underwent mixed fixation, with one knee cemented and the contralateral knee cementless.

To ensure implant homogeneity, the analysis was restricted to two TKA systems from the same manufacturer: the cemented NexGen LPS-Flex and the cementless NexGen Trabecular Metal system (Zimmer Biomet). All other implant systems were excluded. The primary outcome measure was revision of the primary TKA, used as an indicator of implant survival. Information on revision procedures and patient mortality was supplemented using data from the National Arthroplasty Registry of Slovenia (RES) [17], enabling complete ascertainment of outcomes. Revision following primary TKA, therefore, served as the central endpoint for evaluating mid-term implant survival and performance.

All surgical procedures were performed by one of five high-volume orthopedic surgeons, each with a minimum of five years of experience in TKA. Preoperative planning was based on standardized, calibrated anteroposterior radiographs of the entire lower limb. A standard medial parapatellar approach was used in all cases. A uniform surgical technique, fast-track perioperative protocol, and standardized rehabilitation regimen were applied consistently. Restoration of knee alignment was guided by the mechanical axis rather than the anatomical axis [18], a strategy chosen to optimize joint reconstruction, patellar tracking, and balanced flexion–extension gaps in accordance with established surgical principles and the surgeon’s experience [19]. Bone resections were performed manually using conventional intramedullary and/or extramedullary alignment guides, without the use of robotic assistance or computer navigation. Postoperative follow-up was conducted at the same tertiary center, with routine clinical assessments at 3, 6, and 12 months postoperatively, and additional visits arranged as required based on referrals from primary care physicians. This structured follow-up protocol facilitated early detection and management of complications.

The cemented implant configuration consisted of the Zimmer NexGen LPS-Flex and CR-Flex knee prosthesis (Zimmer Biomet), designed to permit flexion of up to 155 degrees [20]. The femoral component is precoated and manufactured from cobalt–chromium–molybdenum (CoCrMo) alloy, selected for its mechanical strength, corrosion resistance, and biocompatibility [21], and incorporates a posterior-stabilized cam-and-post mechanism with an extended anterior flange. The cemented tibial component comprises a precoated titanium alloy (Ti6Al4V) keeled tray combined with a UHMWPE insert.

In the cementless configuration, the NexGen LPS-Flex and CR-Flex femoral component was paired with the NexGen TM Fixed Bearing tibial tray. The femoral component is identical in design and material composition to the cemented version but features a fiber metal mesh surface to promote osseointegration. The TM FB tibial tray is manufactured from titanium alloy and features a TM-coated inferior surface, allowing direct contact with the resected tibial bone, while modular UHMWPE inserts are secured superiorly. The TM coating has a porosity of approximately 70–80%, mimicking cancellous bone architecture and facilitating biological fixation through bone ingrowth [22].

Statistical analysis was performed using SPSS software (version 29.0, IBM Corp., Armonk, NY, USA). Implant survival was analyzed using revision of any component for any reason as one endpoint and revision for aseptic loosening as a separate endpoint. Kaplan–Meier survival curves with 95% confidence intervals were generated, and differences between groups were assessed using the log-rank (Mantel–Cox) test. In patients without revision, survival time was censored at the date of last follow-up, defined as either the date of death or 31 December 2023, whichever occurred first. Categorical variables were reported as absolute values and percentages, whereas continuous variables were expressed as medians with corresponding 95% confidence intervals. Comparisons of categorical data were performed using the chi-square test, and continuous variables were analyzed with the Mann–Whitney U test. A *p*-value of less than 0.05 was considered statistically significant.

## 3. Results

The study included 1202 total knee arthroplasties, of which 826 (68.7%) were cemented, and 376 (31.3%) were cementless. The demographic and clinical features of the patients are summarized in Table 1.

Patients in the cementless group were considerably younger (median age 65.0 years, 95% CI 64.0–66.0) and predominantly male (59%), whereas the cemented group was older (median age 71.0 years, 95% CI 71.0–72.0) and had a higher proportion of females (74.2%) (*p* < 0.001).

During the follow-up period, 94 of 1076 patients (8.7%) died from causes unrelated to their TKA.

**Figure 1:**
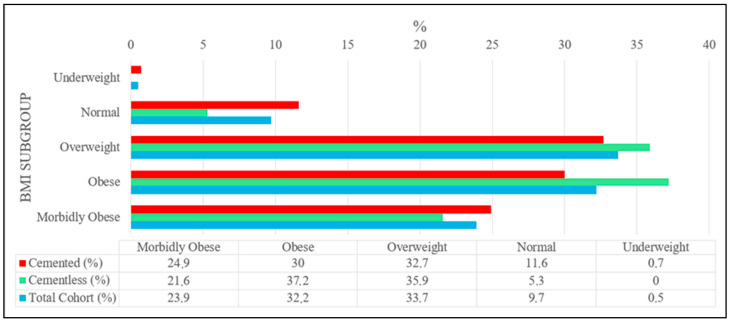
BMI subgroup distribution between cemented and cementless TKA groups.

The BMI categories (underweight < 19 kg/m^2^, normal 19–24.9 kg/m^2^, overweight 25–29.9 kg/m^2^, obese 30–34.9 kg/m^2^, and morbidly obese ≥ 35 kg/m^2^) were distributed similarly between the cemented and cementless groups (*p* > 0.05). Detailed information can be found in Figure 1.

Most patients underwent TKA for primary OA, which was significantly more prevalent in the cementless group (95.2%) than in the cemented group (88.5%) (*p* < 0.001). A higher proportion of patients with rheumatoid arthritis (RA), gout, or psoriatic arthritis received cemented TKAs (*p* = 0.016). The specific indications for TKA are summarized in Table 2.

**Figure 2:**
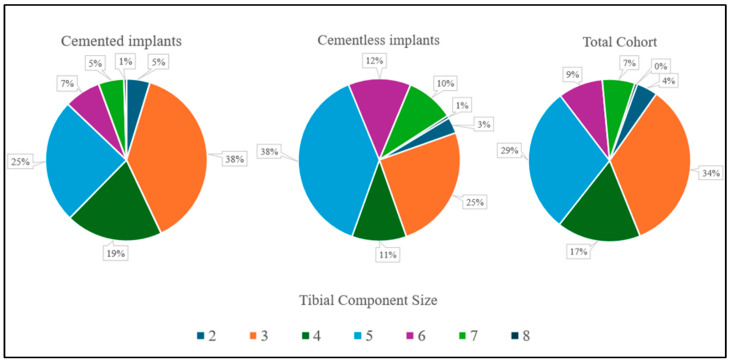
Tibia implant size subgroup distribution between cemented and cementless TKA groups. Note: %—proportion from valid.

PS components were used in most cases (1153; 95.9%), while CR components were implanted in 8 cases (0.97%) in the cemented group and 41 cases (10.9%) in the cementless group (*p* < 0.05). Detailed patient characteristics stratified by implant type are provided in the Supplementary Table S1. Patellar resurfacing was performed in 44 cases (5.3%) in the cemented group and 10 cases (2.7%) in the cementless group (*p* < 0.05). Femoral components ranged from size C to H, tibial components from 2 to 8, and liners from 10 to 20 mm. Tibial implant size distribution, consistent across groups, is shown in Figure 2.

**Figure 3:**
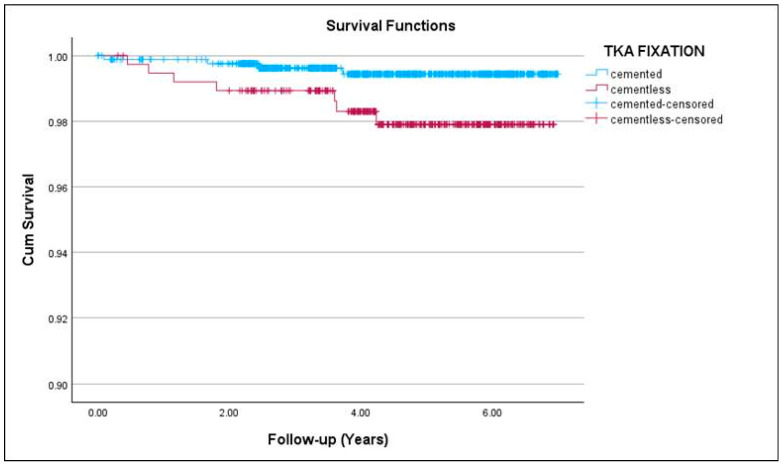
Kaplan–Meier survival curves (95% CI) for cemented and cementless TKAs for aseptic causes of revision.

The 7-year survival rate for all causes was not significantly lower (*p* = 0.63) in the cementless TKA group (97.4%, 95% CI 95.6–99.2) compared to the cemented TKA group (97.8%, 95% CI 96.6–99.0). Revisions for aseptic causes occurred in 7 cementless (1.86%) and 4 cemented (0.84%) TKAs (*p* < 0.05). Notably, tibial component loosening accounted for most of these failures in the cementless group, with 6 of 376 knees (1.59%) revised for aseptic tibial loosening compared with only 2 of 826 knees (0.24%) in the cemented group (*p* < 0.05). Two of the 6 cementless tibial aseptic loosening additionally demonstrated fractured tibial baseplates. The 7-year survival rate for aseptic causes was significantly lower (*p* < 0.05) in the cementless TKA group (97.9%, 95% CI 96.3–99.5) compared to the cemented TKA group (99.4%, 95% CI 98.8–100), as illustrated in Figure 3.

In the multivariable Cox proportional hazards regression analysis adjusted for age, sex, body mass index, and indication for surgery, cementless fixation was associated with a higher risk of aseptic causes of revision compared with cemented fixation (hazard ratio, 2.8; 95% confidence interval, 0.7–10.9), although this difference did not reach statistical significance (*p* = 0.130).

In the cemented tibial TKA group, 15 revisions were performed, compared to 9 in the cementless group. Infection was the main reason for revision in the cemented group, though this difference was not statistically significant, as summarized in Table 3. Median time to revision was 0.69 years (95% CI 0.22–3.83) for cemented and 1.8 years (95% CI 0.45–3.64) for cementless TKAs (*p* > 0.05). For revisions due to aseptic tibial loosening, median times were 3.14 years (95% CI 2.45–3.83) for cemented and 1.48 years (95% CI 0.5–3.64) for cementless TKAs.

## 4. Discussion

To the best of our knowledge, this study represents the largest comparative analysis to date of cementless modular two-pegged TM tibial components in TKA, including 376 cementless and 826 cemented TKAs from the same implant system, with a minimum follow-up of two years. The principal finding was a significantly higher rate of early tibial-sided aseptic loosening in the cementless group compared with the cemented group.

Aseptic tibial loosening has long been recognized as a potential issue with early cementless TKA designs [23,24], though modern implants generally demonstrate loosening rates comparable to those seen with cemented fixation. Supporting this, several studies have reported excellent tibial component stability with cementless designs. For example, a prospective randomized trial by Hannon et al. found no cases of aseptic tibial loosening over six years [25], and Nam et al. similarly observed no aseptic loosening in 80 patients at two years postoperatively [26]. Considering these consistently favorable outcomes, the increased early loosening observed in our study is particularly notable.

Since its introduction in the early 2000s, the TM monoblock tibial component has consistently demonstrated promising stability and clinical outcomes. In a prospective study, Pulido et al. randomized 397 patients to receive conventional modular cemented tibial components, cemented highly porous metal tibial components, or cementless monoblock TM tibial components. After five years, the TM monoblock group exhibited similar fixation, pain relief, and functional outcomes compared with the cemented modular cohort [27]. Ghalayini et al. reported comparable Oxford Knee Scores and KSS at six years in 76 knees with TM monoblock tibial components, with no radiographic evidence of loosening [28]. Similarly, Wojtowicz et al. found that among 339 TKAs with TM monoblock tibial components, no cases required revision for aseptic loosening after a mean follow-up of 8.5 years in patients aged 60 or younger, with 93% of patients expressing overall satisfaction [29]. Hampton et al. reported long-term results from a randomized study of 77 knees comparing cementless TM CR monoblock tibial components with cemented counterparts; over 11–15 years, only one revision occurred in the cementless group, while none were observed in the cemented group, and both implant types demonstrated excellent survivorship [30]. Finally, a 2017 meta-analysis by Hu et al. of six clinical studies including 977 patients found that cementless TM monoblock designs were associated with slightly higher functional scores and fewer radiolucent lines, with no meaningful differences in loosening, revision rates, or postoperative range of motion compared with cemented modular implants over five years [31].

Conversely, Menenghini et al. reported that 8.5% of TM monoblock tibial components exhibited aseptic loosening at 18 months postoperatively in a cohort of 106 patients. Loosening correlated significantly with patient characteristics, particularly taller, heavier, predominantly male individuals, suggesting that increased medial tibial stress in these patients may contribute to early tibial failure [32].

A recent meta-analysis by Li et al., including nine studies published up to July 2023 with 1117 patients (447 cementless, 670 cemented), found no clear long-term clinical or survival advantage for cementless fixation at five or ten years. However, cementless designs were associated with greater preservation of proximal tibial bone density. Notably, seven of the nine included studies evaluated monoblock cementless TM tibial components [33].

Gibian et al. recently conducted a retrospective study comparing revision rates of a two-pegged cementless TM tibial component with its cemented counterpart within the Persona TKA system (Zimmer Biomet, successor to the NexGen Legacy system) at a single high-volume center between 2018 and 2022 [15]. The two-pegged Persona TM tibial baseplate, which had been recalled in 2015, was reintroduced in 2018 following FDA approval of a revised version. The study included 329 cementless and 349 cemented TKAs, with a minimum follow-up of one year and mean follow-up of 1.9 and 2.6 years, respectively. Patients in the cementless group were generally younger and predominantly male. While overall aseptic revision rates did not differ significantly between groups (4.0% cementless vs. 1.7% cemented), tibial aseptic loosening occurred exclusively in the cementless cohort (2.7% vs. 0%), with a mean time to revision of 17.6 months [15]. In contrast, a prospective, multicenter, nonrandomized study by Nakasone et al. reported a 98.6% two-year survival rate of the same two-pegged TM modular tibial design, with no aseptic revisions, although radiolucent lines < 2 mm were observed in 3.4% of patients [34]. Despite differences in follow-up and study design, our findings are consistent with Gibian et al., demonstrating a higher incidence of early tibial-sided aseptic loosening in cementless modular TM components [15].

The largest registry-based comparison of cemented and cementless TKAs within the same system (Zimmer NexGen) utilized data from the Australian Orthopaedic Association National Joint Replacement Registry (AOANJRR) [35]. Cementless non-TM implants had a lower revision risk than cementless TM implants, whereas cementless TM implants demonstrated higher revision rates than cemented implants. Aseptic loosening occurred in 1.0% of cementless TM and 1.2% of cementless non-TM implants. Risk was particularly increased in males and in patients aged 55–74 years. Notably, the registry did not differentiate between monoblock and modular TM designs, and the shorter follow-up for TM implants reflects their more recent introduction. In this context, the higher rate of early tibial-sided aseptic loosening observed in our cementless TM cohort aligns with these registry findings.

Several mechanisms may explain the increased risk of aseptic tibial loosening in cementless modular TM components. The higher stiffness of modular tibial trays, particularly in combination with minor irregularities of the tibial resection—which are more common in younger, active male patients with varus osteoarthritis—can lead to micromotion, instability, bone resorption, and eventual loosening. Such complications may be attenuated by more compliant monoblock designs or cemented fixation. Thermal necrosis from oscillating saw resection, especially in dense or sclerotic bone, may further impair osseointegration and trigger osteolysis [36]. While thermal injury can occur in both cemented and cementless procedures, cementless fixation relies more heavily on the biological integrity of host bone, whereas cemented fixation achieves immediate stability via cement interdigitation. Heat generated during PMMA polymerization is typically localized and has not been strongly associated with clinical failure [37].

To enhance intraoperative flexibility and facilitate insert exchange, a modular successor to the TM monoblock tibial component was developed. However, rare case reports have described early catastrophic failures due to incomplete biological ingrowth [38]. Previous work by Fokter et al., using retrieval analysis combined with histology, micro–Proton-Induced X-ray Emission (micro-PIXE), and finite-element modeling, suggests that insufficient osseointegration and micromotion at the tibial bone–implant interface may play a central role in the development of aseptic loosening of trabecular metal modular tibial components [39]. These analyses demonstrated limited bone ingrowth, particularly on the medial side, together with the presence of tantalum and titanium wear debris in the surrounding tissue, indicating ongoing micromotion and a local biological response at the interface. Such micromotion may be amplified by higher mechanical loads in younger, more active, and predominantly male patients, while the specific two-peg design of the tibial baseplate may be insufficient to adequately counteract these forces. In combination with bone loss potentially induced by metal wear debris, these factors may establish a self-reinforcing cycle of increasing micromotion, progressive loss of fixation, and eventual aseptic loosening of the tibial component. However, early and mid-term outcomes of the modular TM design have generally been favorable [13,40,41,42,43,44]. Meta-analyses of Level I studies have demonstrated comparable survivorship and complication rates between monoblock and modular designs, with modular implants showing slightly superior functional outcomes and stability on RSA at short-term follow-up [45]. It has been shown that monoblock two-pegged TM tibial trays preserve periprosthetic bone mineral density more effectively than cemented implants and in a pattern comparable to the nonoperative control limb [9]. However, long-term follow-up studies have not demonstrated a sustained protective effect of monoblock TM tibial components on proximal tibial bone loss after total knee arthroplasty [46]. Importantly, comparable periprosthetic BMD or long-term RSA data are lacking for modular two-pegged TM tibial designs. In concordance with the findings of Gibian et al., our results indicate that the addition of modularity to an uncemented two-pegged TM tibial component does not confer a survival advantage in total knee arthroplasty [15].

This study represents one of the largest series comparing cementless modular TM tibial trays with equivalent cemented designs, with similar overall follow-up durations. Revision and mortality data were complete for all patients, supported by the near-complete coverage of RES [17]. Nevertheless, several limitations should be acknowledged. First, the retrospective design and mid-term follow-up limit generalizability. Second, cementless TKAs were predominantly performed in younger, more active male patients, introducing potential selection bias. Third, both PS and CR designs were included, along with knees with or without patellar resurfacing, which may influence outcomes. Fourth, all procedures involved high-flexion implants, so results may not be directly applicable to standard TKA designs. Although high-flexion implants can provide a modest increase in postoperative range of motion, prior studies have shown no significant differences in functional outcomes, satisfaction, or complication rates compared with standard designs [47,48], although some long-term reports suggest a higher incidence of radiolucent lines and revisions without clear clinical benefit [49]. Fifth, patients were not routinely followed with standardized postoperative radiographs. Consequently, radiographic findings such as radiolucent lines, component subsidence, or implant migration could not be systematically assessed, limiting the ability to identify early failure patterns and to more precisely determine the mechanisms underlying aseptic loosening. Finally, patients were not routinely followed with standardized postoperative radiographs. As a result, radiographic signs such as radiolucent lines, component subsidence, or implant migration could not be systematically assessed, limiting the ability to identify early failure patterns and to more precisely determine the mechanisms underlying aseptic loosening. Prospective studies incorporating these factors are needed.

## 5. Conclusions

In summary, cementless modular TKAs with two-pegged TM tibial components demonstrated inferior mid-term outcomes compared with their cemented counterparts, particularly with respect to aseptic tibial loosening. These findings underscore the need for careful patient selection and highlight the ongoing necessity for innovation in implant design to improve long-term survivorship and clinical outcomes in total knee arthroplasty.

## Figures and Tables

**Table 1:** Demographic and clinical characteristics of the study cohort.

Parameter	Cemented	Cementless	Total Cohort	*p*-Value
Number of TKA implanted (N (%))	826 (68.7)	376 (31.3)	1202 (100.0)	/
Gender (Male/Female; N (%))	213/613 (25.8/74.2)	222/154 (59.0/41.0)	435/767 (36.2/63.8)	<0.001
Age at Implantation (years; median and 95% CI)	71.0 (71.0–72.0)	65.0 (64.0–66.0)	69.0 (68.0–70.0)	<0.001
Side (Left/Right; N (%))	411/415 (49.8/50.2)	186/190 (49.5/50.5)	597/605 (49.7/50.3)	0.950
BMI (kg/m^2^; median and 95% CI)	30.0 (30.0–31.0)	31.0 (30.0–31.0)	30.0 (30.0–31.0)	0.234

Note: N—count; %—proportion from valid; TKA—total knee arthroplasty; CI—confidence interval. Data on weight were not available for 222 (18.5%) knees, and BMI data were missing for 222 (18.5%) knees.

**Table 2:** Indications for primary total knee arthroplasty.

Indication	Cemented (N (%))	Cementless (N (%))	Total Cohort (N (%))	*p*-Value
Primary Osteoarthritis	731 (88.5)	358 (95.2)	1089 (90.6)	<0.001
RA/Gout/Psoriatic Arthritis	29 (3.5)	4 (1.1)	33 (2.7)	0.016
Fracture	15 (1.8)	3 (0.8)	18 (1.5)	0.177
Torn Ligaments	11 (1.3)	2 (0.5)	13 (1.1)	0.366
Infection	4 (0.5)	0 (0.0)	4 (0.3)	0.316
Avascular Bone Necrosis	5 (0.6)	0 (0.0)	5 (0.4)	0.333
Other	19 (2.3)	4 (1.1)	23 (1.9)	0.146
No Data	12 (1.5)	5 (1.3)	17 (1.4)	/

Note: N—count; %—proportion from valid; RA—rheumatoid arthritis; Data for the indication for primary TKA was not provided for 17 knees (1.4%).

**Table 3:** Indications for revision surgery.

Indication	Cemented (N, (%))	Cementless (N, (%))	Total Cohort (N, (%))	*p*-Value
Loosening of the Tibial Component	2 (13.3%)	6 (66.7%)	8 (33.3%)	0.021
Early Infection (<3 months)	4 (26.7%)	0 (0.0%)	4 (16.7%)	0.259
Late Infection (>3 months)	7 (46.6%)	2 (22.2%)	9 (37.5%)	0.389
Dislocation of the Patella	1 (6.7%)	0 (0.0%)	1 (4.2%)	1.000
Instability of Collateral Ligaments	1 (6.7%)	1 (11.1%)	2 (8.3%)	1.000
Total	15 (62.5%)	9 (37.5%)	24 (100.0%)	0.507

Note: N—count; %—proportion from valid.

## Data Availability

Datasets are available on request from the authors.

## Supplementary Materials

The following supporting information can be downloaded at https://www.mdpi.com/article/10.3390/medicina62020296/s1. Table S1. Baseline demographic and clinical characteristics according to implant design (PS and CR).

## Abbreviations

The following abbreviations are used in this manuscript:

TKA	Total knee arthroplasty
PS	Posterior-stabilized
CR	Cruciate retaining
OA	Osteoarthritis
BMI	Body mass index
PMMA	Polymethylmethacrylate
RSA	Radiostereophotogrammetric analysis
UHMWPE	Ultrahigh-molecular-weight polyethylene
BMD	Body mineral density
KSS	Knee Society Score
WOMAC	Western Ontario and McMaster Universities Osteoarthritis Index
AP	Anteroposterior
XHLPE	Highly cross-linked polyethylene
TM FB	Trabecular Metal Fixed Bearing
RA	Rheumatoid arthritis
OKS	Oxford Knee Scores
AOANJRR	Australian Orthopaedic Association National Joint Replacement Registry
