# Supplementary material for: Two-Peg Cementless Trabecular Metal Modular Tibial Components in Total Knee Arthroplasty: A Single-Center Comparative Study with Cemented Counterparts
Source: Medicina (Kaunas). 2026 Feb 2;62(2):296. doi: 10.3390/medicina62020296 (PMC12942213; doi:10.3390/medicina62020296)
Supplement: Supplementary file 1 [file medicina-62-00296-s001.zip › medicina-4111602-supplementary.pdf]

Supplementary Material

Table S1. Baseline demographic and clinical characteristics according to implant design (PS and CR).

Parameter	PS	CR	Total Cohort	p-Value
**Number of TKA implanted (N (%))**	1153 (95.9)	49 (4.1)	1202 (100.0)	/
**Fixation (Cemented/Cementless; N (%))**	818/335 (70.9/29.1)	8/41 (16.3/83.7)	826/376 (68.7/31.3)	< 0.001
**Gender (Male/Female; N (%))**	404/749 (35.0/65.0)	31/18 (63.3/36.7)	435/767 (36.2/63.8)	< 0.001
**Age at Implantation (years; mean and 95% CI)**	69.0 (68.5–69.6)	64.0 (61.4–66.5)	69.0 (68.0–70.0)	< 0.001
**Side (Left/Right; N (%))**	575/578 (49.9/50.1)	22/27 (44.9/55.1)	597/605 (49.7/50.3)	0.950
**BMI (kg/m^2; mean and 95% CI)**	31.0 (30.6–31.3)	29.7 (28.0–31.3)	30.0 (30.0-31.0)	0.144

Note: N–count; %–proportion from valid; TKA–total knee arthroplasty; CI–confidence interval; PS–posterior stabilized; CR–Cruciate retaining. Data on weight were not available for 222 (18.5%) knees, and BMI data were missing for 222 (18.5%) knees.
